# Objective Assessment of Regional Stiffness in Vastus Lateralis with Different Measurement Methods: A Reliability Study

**DOI:** 10.3390/s21093213

**Published:** 2021-05-06

**Authors:** Alfredo Bravo-Sánchez, Pablo Abián, Jorge Sánchez-Infante, Paula Esteban-Gacía, Fernando Jiménez, Javier Abián-Vicén

**Affiliations:** 1 Performance and Sport Rehabilitation Laboratory, Faculty of Sport Sciences, University of Castilla-La Mancha, 45071 Toledo, Spain; alfredo.bravo@uclm.es (A.B.-S.); jorge.fisio.uclm@gmail.com (J.S.-I.); paula.esteban@uclm.es (P.E.-G.); josefernando.jimenez@uclm.es (F.J.); 2 Faculty of Humanities and Social Sciences, Comillas Pontifical University, 28049 Madrid, Spain; pabian@comillas.edu

**Keywords:** shear wave elastography, strain elastography, MyotonPRO, tensiomyography, mechanical properties

## Abstract

The objective of this study was to evaluate the reliability of four methods of assessing vastus lateralis (VL) stiffness, and to describe the influence of structural characteristics on them. The stiffness of the dominant lower-limb’s VL was evaluated in 53 healthy participants (28.4 ± 9.1 years) with shear wave elastography (SWE), strain elastography (SE), myotonometry and tensiomyography (TMG). The SWE, SE and myotonometry were performed at 50%, and TMG was assessed at 30%, of the length from the upper pole of the patella to the greater trochanter. The thickness of the VL, adipose tissue and superficial connective tissue was also measured with ultrasound. Three repeated measurements were acquired to assess reliability, using intraclass correlation coefficients (ICC). Pearson’s correlation coefficients were calculated to determine the relationships between methodologic assessments and between structural characteristics and stiffness assessments of the VL. Myotonometry (ICC = 0.93; 95%-CI = 0.89,0.96) and TMG (ICC = 0.89; 95%-CI = 0.82,0.94) showed excellent inter-day reliability whereas with SWE (ICC = 0.62; 95%-CI = 0.41,0.77) and SE (ICC = 0.71; 95%-CI = 0.57,0.81) reliability was moderate. Significant correlations were found between myotonometry and VL thickness (r = 0.361; *p* = 0.008), adipose tissue thickness (r = −0.459; *p* = 0.001) and superficial connective tissue thickness (r = 0.340; *p* = 0.013). Myotonometry and TMG showed the best reliability values, although myotonometry stiffness values were influenced by the structural variables of the supra-adjacent tissue.

## 1. Introduction

Stiffness is a mechanical property of muscles in relation to the rate of force development, and a positive relation between stiffness and fast stretch shortening cycle activity performance has been described in previous research [1]. In addition, higher values of stiffness have also been related to higher injury risk [2]. Therefore, the study of muscle stiffness should be a priority in sport and even in the public health area. Different acquisition methods have been employed to assess muscle stiffness with good reliability [3,4,5,6], although the relations among them should be clarified to avoid confusion in their interpretation.

Elastography ultrasound is widely used to quantify the mechanical properties of tissues [5,7,8,9]. There are different methods of elastography which have been applied to describe passive muscle stiffness based on the adaptation of the tissue to mechanical stimuli. In this line, the small time shift caused by the mechanical stimuli and the tissue deformation are employed to calculated the stiffness of the tissue under the probe [10], the mechanical stimuli being different between the types of elastography. Shear wave elastography (SWE) uses the propagation of shear waves and their velocity to provide shear modulus (Kpa) [11], while strain ratio elastography (SE) reports the elastography index after light repetitive compression performed with a hand-held transducer [12]. Despite elastography studies having shown good reliability for measuring passive muscle and tendon stiffness [5,13], there are several limitations, such as saturation of the elastogram, anisotropy and operator dependence [14,15,16], which made it necessary to develop valid and reliable elastography assessment protocols. Furthermore, previous studies showed a good relationship between SWE and SE for the diagnosis of tumours [17], although any potential application in the study of muscles should be evaluated.

The MyotonPRO has been developed as a simple and reliable tool that requires less technical expertise for the assessment of muscle mechanical properties than elastography measurements [5]. The measuring principle of the MyotonPRO is that the oscillations caused by multiple short pulses from a test probe, and their shapes, reflect the visco-elastic properties of the tissue such as tone and dynamic stiffness [18]. Dynamic stiffness characterises muscle resistance to an external force that changes its initial shape [18]. In addition, dynamic stiffness showed a positive correlation with shear modulus measured with SWE for the gastrocnemius and Achilles tendon [7], which should be corroborated in studies with a larger sample size. Finally, due to the data acquisition system, large values of adipose tissue or superficial connective tissue could affect the validity and reliability of the measurements [19]. Another option to estimate muscles stiffness is tensiomyography (TMG), commonly used to assess the contractile properties of muscles and described as an easy to apply technique for stiffness measurement [6]. TMG allows non-invasive and reliable [6,20,21] assessment of muscle belly enlargement in the transversal plane during an isometric twitch contraction by means of a digital, high-precision displacement sensor which assesses the muscle’s mechanical contractile responses. As occurs with MyotonPRO, TMG uses a slight pre-tension that the displacement sensor exerts on the muscle belly before the contraction [20]. In TMG analysis, stiffness is indirectly calculated and interpreted from maximal radial displacement (Dm), with lower values related to stiffer tissues [20], as the muscle contraction is the mechanical stimuli employed to calculate stiffness. TMG showed similar values of reliability compared to the MyotonPRO in previous research [22], although the relations between TMG, MyotonPRO and elastography measurements—SWE and SE—have been less studied.

The present study aims to evaluate the reliability of SWE, SE, the MyotonPRO and TMG to assess vastus lateralis muscle stiffness and to establish their relations. Secondly, we aimed to study the influence of muscle structural characteristics on stiffness evaluations.

## 2. Materials and Methods

### 2.1. Participants and Inclusion Procedure

Fifty-three recreational and physically active volunteers were recruited in the study (thirty-three men and twenty women; age: 28.3 ± 9.1 years; height: 173.0 ± 7.5 cm; weight: 69.5 ± 9.8 kg; daily physical activity: 1.7 ± 1.6 h·day^−1^). All subjects signed an informed consent form that was previously approved by the Ethics Committee of Clinical Research at the Toledo Hospital complex. The participants with an injury or any pain that would prevent them from doing their usual physical activity were excluded from the sample. All data were recorded on the dominant lower limb, which was indicated by the participants before data collection, via a questionnaire, as the self-reported preferred kicking lower limb [23].

### 2.2. Design and Procedure

A repeated measures design was carried out to determine the intra-day and inter-day reliability. Each participant undertook the testing procedure, composed by four stiffness measurements, on three different sessions, with one-hour (intra-day reliability) and one-day (inter-day reliability) rest intervals between testing sessions. The evaluator was blinded to the test results from previous testing sessions.

The structural characteristics of the vastus lateralis (VL) were studied with a Logiq^®^ S8 ultrasound (GE Healthcare, Milwaukee, WI, USA) with an 8–12 MHz multi frequency linear probe (ML6–15-D; GE Healthcare system) after a 5 min rest in a supine position [24].

The mechanical properties of the VL were assessed after the structural variable assessment with two types of elastography, SWE and SE. In addition, mechanical properties were evaluated with a hand-held Myoton^®^ Pro myotonometer (Myoton AS, Tallinn, Estonia). Finally, the contractile properties of the VL were measured with a TMG device (BMC-Ltd., Ljubljana, Slovenia).

**Figure 1:**
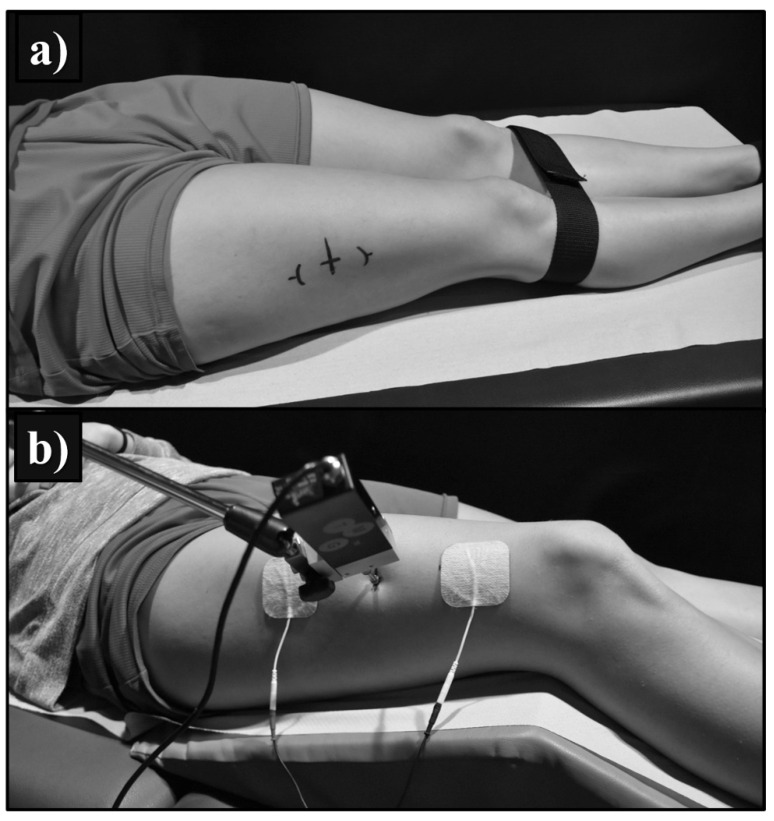
Position of participants during assessments. (**a**) Measurement position of ultrasound and MyotonPRO exams of Vastus Lateralis. (**b**) Measurement position of tensiomyography exam of Vastus Lateralis.

The anatomical localisation of the measurement points and participants’ positions were standardised for all subjects and established according to previous studies [4,20,25]. Ultrasound and MyotonPRO exams were performed at the midpoint from the upper pole of the patella to the greater trochanter [4,25], with the participants in a supine position and with the knee fully extended (Figure 1). The TMG measurement point was located at 30% of the length from the upper pole of the patella to the greater trochanter [20] with the participant in a supine position with 30^o^ of knee flexion using a foam pad for leg support (Figure 1). Their position was controlled with the same foam support at the level of the feet as was used to prevent external rotation of the leg during the elastography exam. To prevent the possibility of circadian effects, the participants attended the laboratory for data collection at the same time of day on the first and second day of measurements.

### 2.3. Ultrasound Measurements

**Figure 2:**
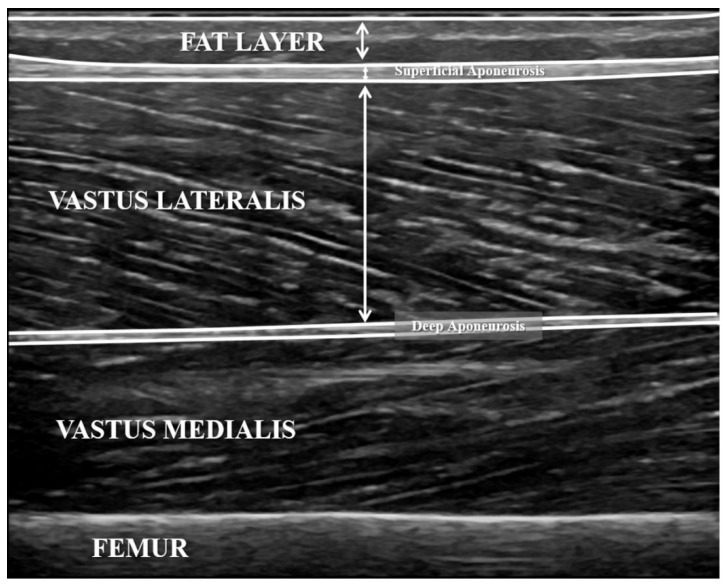
B-mode ultrasound exam of vastus lateralis. Vastus lateralis thickness was calculated as the distance in mm between the deep edge of the superficial aponeurosis and the superficial edge of the deep aponeurosis; adipose tissue thickness was calculated as the distance in mm between the skin and the superficial edge of the superficial aponeurosis; superficial connective tissue thickness was calculated as the distance in mm between the deep edge and the superficial edge of the superficial aponeurosis.

For the structural analysis of the VL, the patient was in a supine position with the knees fully extended [24]. An experienced certified musculoskeletal ultrasound sonographer carried out all exams and analysed the US data. The ultrasound probe was aligned with the fascicle direction [24] to measure the thickness of the VL (distance in mm between the deep edge of the superficial aponeurosis and the superficial edge of the deep aponeurosis), adipose tissue thickness (distance in mm between the skin and the superficial edge of the superficial aponeurosis) and superficial connective tissue thickness (distance in mm between the deep edge and the superficial edge of the superficial aponeurosis) [26] (Figure 2).

### 2.4. Elastography Measurements

Both elastography exams, SWE and SE, were performed with a Logiq^®^ S8 ultrasound (GE Healthcare, Milwaukee, WI, USA) with an 8–12 MHz multi frequency linear probe (9L-D; GE Healthcare system). The probe was placed with very light pressure on top of the gel and aligned with the VL fascicles (longitudinal scan). The B-mode image and elastogram were displayed side-by-side on the screen during elastography measurements, and to avoid anisotropy the following criteria were applied: (a) the examination probe was held perpendicular to the tissue; (b) the B-mode image showed continuous striations (muscle fascicle) extending from superficial to deep aponeurosis [27]. To maximise inter-day reliability and minimise the duration of transducer re-positioning at the same location during subsequent sessions on each subject, 4 permanent waterproof skin landmarks were drawn with a marker under 2D-mode monitoring during the pre-session [14]. One line was drawn 15 to 20 cm above the upper edge of the patella parallel to the longitudinal axis of the centre of the VL. Three additional lines were drawn at both sides of the probe and its midpoint to identify the position of the probe during the first measurement [14]. The region of interest (ROI) was set at 10 mm in diameter for SWE and SE exams [11]. The ROI was placed within the muscle, below the superficial aponeurosis, at a depth lower than 40 mm to avoid the influence of depth on shear wave transmission [11].

**Figure 3:**
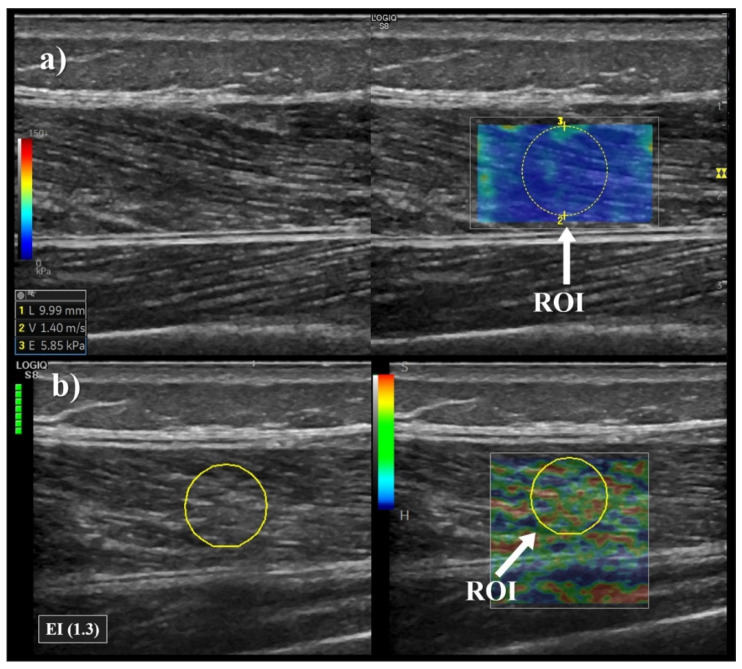
Example of elastography measurement of Vastus Lateralis. The measurement point was placed at 50% of the distance from the upper edge of the patella bone to the greater trochanter. (**a**) Shear wave measurement of Vastus Lateralis; E = shear modulus. (**b**) Strain elastography measurement of Vastus Lateralis; EI = Elastography Index. ROI = region of interest.

For SWE, the shear wave modulus was evaluated after 5-second acquisition exams when the real time colour map was as homogeneous as possible. Five consecutive SWE exams were performed and their mean value was described as the shear modulus (kPa) of the VL (Figure 3a), and was employed to evaluated the stiffness of the VL with SWE. The SWE system reads and quantifies the propagation velocity of the shear waves generated, transforming it into KPa values through the Young’s modulus formula, with the assumption that the elastic response of the tissue is linear and the tissue density is always 1000 kg·m^−3^ [28].

The SE exam was performed by applying light repetitive compression with the hand-held transducer. The amount and uniformity of compression were standardised using a pressure graph that appears on the screen. The best cine image derived from at least three compression-relaxation cycles was used for the assessment of the elastography index (EI) [12]. The EI was calculated automatically by the SE machine by comparing the muscle to the adjacent tissues, with higher values on the EI related to a higher stiffness level [2]. The colour code indicated the relative stiffness of the tissues within the region of interest and was translated into a numerical score from 1 to 6 (1 indicates very soft tissues and 6 very stiff tissues) as follows: red = 1 (softest); orange = 2; yellow = 3; green = 4; light blue = 5; blue = 6 (hardest). The mean of three consecutive measurements was used to describe the EI of the VL (Figure 3b).

### 2.5. MyotonPRO Measurements

The MyotonPro (MytonPro, Myoton Ltd.s., Tartu, Estonia) is a portable device employed to assess the deformation properties of natural damped oscillations produced by a short (15 ms) mechanical tap on the surface of the skin that shows good reliability for lower-limb muscles [3]. The probe of the MyotonPRO was placed perpendicular and was compressed to the skin until the light of the device turned green. Ten short (15 ms) mechanical impulses (0.4 N) were generated via an electromagnetic mechanism after precompression of the subcutaneous tissues and recorded by the device’s accelerometer [18]. Computational software creates data on dynamic stiffness (N/m) of the underlying tissue, based on equations calculated from the acceleration of the testing probe during oscillations [18]. Dynamic stiffness reflects the resistance of the tissue to the force that changes its shape and higher values of dynamic stiffness imply that more energy is needed to modify the shape of the tissue [18]. The mean of three consecutive measurements was used to describe dynamic stiffness of the VL which indicates the tissue resistance to a contraction or to an external force that deforms its initial shape.

### 2.6. Tensiomyography Measurements

The oscillations of the muscle belly in response to an electrically-induced twitch were recorded on the skin’s surface using a sensitive displacement sensor (TMG–BMC, Slovenia). The sensor was set perpendicular to the skin, overlying the muscle belly at the point described above. A single 1-ms maximal monophasic electrical impulse (30 Volts with amplitude ranging from 60 to 100 mA) through the cathode and the anode, that were 5 cm distally and 5 cm proximally to the measuring point, respectively, was used to elicit a twitch. The electric pulse amplitude started at 30 mA and was increased by 10 mA until maximal radial displacement (Dm) was reached. A 30 s resting period was applied between electrical stimuli to avoid potentiation effects [29]. The Dm defined as the peak amplitude in the displacement–time curve of the tensiomyographical twitch response was used to describe VL stiffness [21].

### 2.7. Statistical Analysis

The statistical analysis was performed with IBM SPSS Statistics 24.0 (SPSS, Chicago, Illinois). All data were expressed as mean and standard deviation. The data were tested for normality with a Kolmogorov–Smirnov test. Since the assumption of normality (all variables *p* > 0.05) was verified, the test-retest reliability was estimated using Intraclass Correlation Coefficients (ICCs) to assess the intra-day (set 1 vs. set 2) and inter-day reliability (set 1 vs. set 3) [30]. In addition, a paired sample t-test was performed to identify the change in the mean between intra-day (set 1 vs. set 2) and inter-day (set 1 vs. set 3) values. Reliability thresholds for ICC values were defined as poor (<0.50), moderate (0.50–0.75), good (0.75–0.90), and excellent (>0.90) [31]. Bland–Altman plots were also used to measure inter- and intra-operator reliability to visualise the degree of agreement and to identify systematic bias. Standard error of measurement (SEM) and minimal detectable change (MDC) were calculated as follows: SEM = SDdiff/√2, and MDC = SEM × 1.96 × √2, where SDdiff is the standard deviation (SD) of the difference scores [32]. Only differences between two measurements that exceed the MDC represent a real (non-error) change in scores for a subject [32]. Pearson’s correlation coefficients were calculated to determine the relationships between methodologic assessments and between structural characteristics and mechanical properties of the VL. The level of significance was determined as *p* < 0.05.

## 3. Results

### 3.1. Test-Retest Reliability

Paired t-test analyses reported no significant differences between set 1 and set 2 (intra-day) or between set 1 and set 3 (inter-day) in all variables analysed for describing VL mechanical and contractile properties (*p* > 0.05) (Table 1). SWE showed a good intra-day reliability (ICC = 0.80 with a CV of 15.3%; SEM = 3.17 Kpa) and a moderate inter-day reliability (ICC = 0.62 with a CV of 33.8%; SEM = 5.60 Kpa) for VL shear modulus. Moderate intra-day (ICC = 0.71 with a CV of 11.8%; SEM = 0.26 A.U.) and inter-day (ICC = 0.71 with a CV of 13.0%; SEM = 0.27 A.U.) reliability was also exhibited by SE. The intra-day reliability was excellent for the MyotonPRO (ICC = 0.97 with a CV of 3.3%; SEM = 10.24 N/m) and TMG (ICC = 0.91 with a CV of 7.7%; SEM = 0.54 mm). In addition, excellent reliability was described for the MyotonPRO (ICC = 0.93 with a CV of 3.6%; SEM = 14.57 Nm) and TMG (ICC = 0.89 with a CV of 9.5%; SEM = 0.56 mm) inter-day measurements. (Table 1). The intra-day and inter-day MDC values are described in Table 1.

### 3.2. Bland-Altman Results

**Figure 4:**
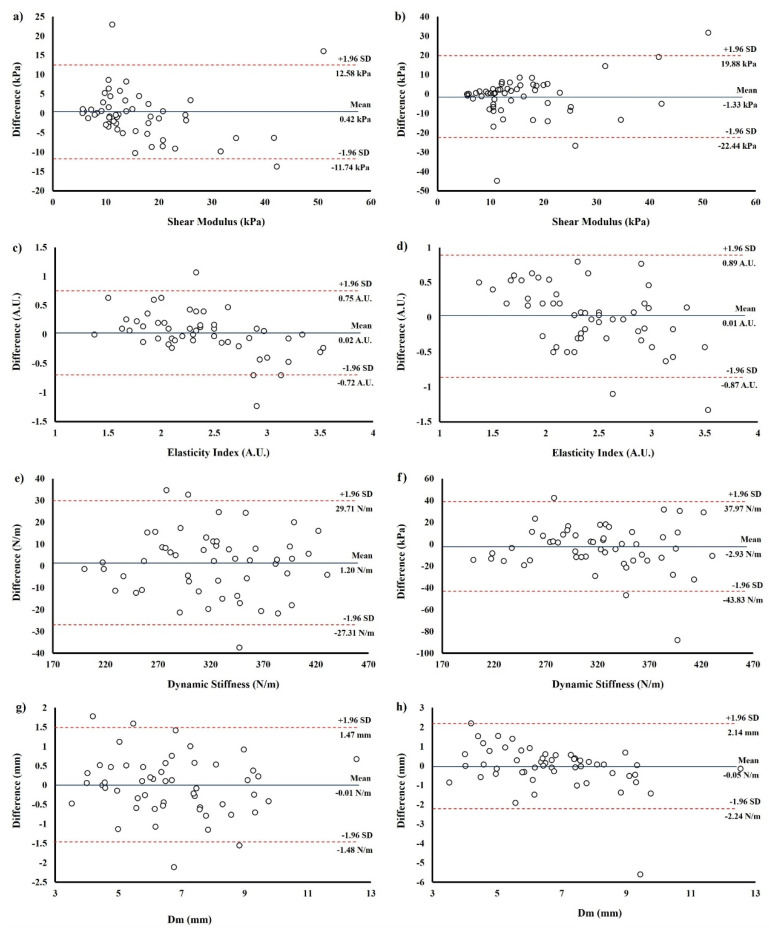
Bland-Altman plots of intra- and inter-rater reliability of Vastus Lateralis (VL) stiffness. (**a**,**b**) Intra- and inter-rater reliability of VL shear modulus measured with shear wave elastography. (**c**,**d**) Intra- and inter-rater reliability of VL elastography index measured with strain elastography. (**e**,**f**) Intra- and inter-rater reliability of VL dynamic stiffness measured with the MyotonPRO. (**g**,**h**) Intra- and inter-rater reliability of VL maximal radial displacement measured with tensiomyography.

The Bland–Altman plot for VL mechanical properties shows the bias line, or the mean difference in VL stiffness intra-day and inter-day for SWE, SE, MyotonPRO and TMG assessments. The Bland-Altman plot shows no systematic bias, as the data points are distributed equally below and above the mean for all equipment at both measurement moments (Figure 4).

### 3.3. Correlation Results

After B-mode ultrasound analysis, VL structural values were (mean ± SD): VL thickness = 19.40 ± 0.42 mm; Adipose tissue thickness = 6.67 ± 3.39 mm; and superficial connective tissue thickness = 1.40 ± 0.45 mm. Pearson’s correlation revealed a positive relation between shear modulus measured with SWE and EI assessed with SE (r = 0.572; *p* < 0.001). In addition, higher values of the VL thickness showed a positive correlation with dynamic stiffness measured with the MyotonPRO (r = 0.361; *p* = 0.008), as occurred with the thickness of connective tissue and dynamic stiffness assessed with the MyotonPRO (r = 0.340; *p* = 0.013). Finally, a negative correlation was described between MyotonPRO dynamic stiffness and the thickness of the adipose tissue (r = −0.459; *p* = 0.001). No significant correlations were found between structural variables and SWE, SE and TMG exams (*p* > 0.05).

## 4. Discussion

The present study shows the feasibility of different methods used to assess the stiffness of the VL. We found excellent intra- and inter-day reliability for evaluating VL stiffness in healthy participants, with a relatively low SEM and MDC with the MyotonPRO and TMG. In addition, we found moderate and good intra-day and moderate inter-day reliability for SE and SWE exams and also a positive correlation between both methods. No other correlation was found between elastography techniques and the MyotonPRO and TMG, which might suggest that elastography techniques assess a different type of stiffness from the MyotonPRO and TMG. Finally, the influence of structural variables in myotonometry assessment was also described after correlation analysis: MyotonPRO exams showed a negative correlation with adipose tissue thickness and showed a positive correlation with superficial connective tissue thickness. Therefore, the structural variables should be considered as research bias in the measurements carried out with the MyotonPRO.

The intra- and inter-day reliability of SWE described in this study (ICC = 0.62 to 0.80) was similar to what previous investigations have reported in healthy participants [11,13], although the CV registered in our study (CV = 15.3 to 33.8%) was higher in both measurement time comparisons [11,13]. SWE is based on the interpretation of the propagation of shear waves to estimate shear wave velocity (m/s) and shear modulus (kPa) [33], so factors such as depth, the anatomical structures evaluated, or the degree of stenosis could affect the results and reliability tasks [8,9,33,34]. The thickness of superficial connective tissue could provoke the saturation of the elastogram, which occurs when the elasticity of the tissue exceeds the upper limit of the elasticity scale that is used to render the elastogram, and would make the SWE exam of subjacent tissue difficult [16]. In our study, the B-mode exam showed that the superficial connective tissue was very thick and in some participants two layers appeared well differentiated, where the most superficial layer corresponded to the iliotibial band and the deep layer was the superficial aponeurosis of the VL (Figure 2). In this vein, higher ICCs and lower CVs for SWE were described in previous studies which evaluated muscles without the influence of connective tissue such as the gastrocnemius medialis [13] and rectus femoris [35]. In addition, significant differences were found between muscle belly and myotendinous junction shear modulus [36]. Therefore, future studies should establish the relation between the shear modulus and connective tissue thickness, to standardise the SWE interpretation.

The saturation of the elastogram could be one of the main factors which make the SWE exams difficult [16]. Anatomical points with thick superficial connective tissue could present problems for analysing the shear wave velocity, similarly to measurements made next to the bone [37], due to the higher stiffness of myotendinous junctions and fascia [28,36]. SE techniques assess the stiffness of the tissue thought the EI value which describes the adaptation of the tissue to repetitive compression with a hand-held device [2]. This study is the first to report moderate reliability (ICC = 0.71) of the EI applied to assess the VL in healthy participants according to previous research carried out on the patellar and quadriceps tendons [3,15]. In addition, despite SE having higher operator dependency than SWE exams [28], SE and SWE showed a positive correlation, and lower CV were obtained for SE compared to SWE assessment. Furthermore, previous studies concluded that experienced examiners could obtain good reliability values for SE [5]. Therefore, SE exams made by experienced examiners should be used prior to SWE for the stiffness assessment in regions where the saturation of the elastogram could be a limitation, as occurs in participants with thicker conjunctive tissue.

SWE’s and SE’s high cost and operator dependence [38] may reduce their applicability in ecological environments. In this context, the MyotonPRO and TMG measurements may become relevant. These instruments have exhibited excellent intra- and inter-day reliability (ICC = 0.93 to 0.99) with a low CV (3.3 to 9.5%), according to previous studies which evaluated the stiffness of the vastus medialis and vastus lateralis [4,6,39]. The dynamic stiffness measured with the MyotonPRO is defined as the resistance of the tissue to deformation when an external force is applied [18], similar to SE evaluation which analyses the tissue adaptation to an external force applied with the transducer for describing the stiffness [2]. The force applied by the MyotonPRO (0.4 N) was probably lower than in SE exams, which could imply that the former assesses mainly the properties of the superficial layers of adipose, connective and muscle tissue [40] whereas SE, which applies a larger mechanical stimulus, could also assess the deeper muscle tissue. This fact was previously showed by Mustalampi et al. [40], who compared the MyotonPRO and the Computerised Muscle Tonometer. On the other hand, TMG defines the stiffness as the peak amplitude in the displacement–time curve after electrical stimuli [6,20], and, as occurs with SE exams, could have more effectivity for deeper muscle tissue assessment than the MyotonPRO, although future studies are warranted to confirm this supposition. Finally, the MyotonPRO and TMG might represent a non-invasive and cost-effective alternative for quantifying the stiffness of the VL. Finally, the lack of descriptive studies on VL stiffness in healthy patients makes MDC the reference variable to detect significant changes in VL stiffness. In this line, an increase in shear modulus, EI or dynamic stiffness greater than MDC will indicate an increase of the muscle stiffness. For TMG assessment, a significant increase in stiffness will be interpreted as a reduction in Dm values above the MDC described in our study.

After correlational study, it is likely that the myotonometric measures are influenced by structural variables such as adipose, connective tissue, and muscle thickness. We found that the dynamic stiffness measured with the MyotonPRO showed a negative correlation with adipose tissue thickness and a positive relation with superficial connective tissue thickness. The MyotonPRO assessment is based on the acceleration of the testing probe during oscillations [18], so the characteristic of suprajacent tissue (adipose and connective tissue) could influence the assessment [40]. Furthermore, Feng et al. [7] found a positive correlation between SWE and the MyotonPRO for mechanical property evaluation of the rectus femoris, but we did not find any interaction between SWE or SE and MyotonPRO measurements. This could be because the thickness of superficial conjunctive tissue of the rectus femoris would be lower than VL superficial conjunctive tissue, although data on structural variables were not provided. Therefore, future studies should establish the maximal thickness of suprajacent tissue that does not influence myotonometry exams. Despite the term stiffness being commonly used to define the viscoelastic characteristics of the assessed tissue and SWE and SE showing a positive correlation, no other correlation was found between the assessment methods, which might suggest that elastography techniques assess a different type of stiffness from the MyotonPRO and TMG, and future studies should define stiffness in relation to the measurement technique used.

There are several limitations to this study. First, although our team suggested that participants should be relaxed during the whole experiment, muscle contraction could not be precisely controlled without the use of electromyography. Second, the measurements of SWE and SE were made with a single ultrasound equip (LogiqS8) so the mean values of shear modulus and EI should only be compared with this equipment because of the variations among different machines [11]. In addition, elastography technique assumes that underlying tissue is isotropic, elastic and locally homogenous [10], so the results obtained in this study should be corroborated in others points of muscle belly. Third, TMG measurements were made following previous protocols [20] at a different point from the SWE, SE and the MyotonPRO; therefore, future investigations at the same muscle points are warranted to analyse within-methods correlations. Finally, we evaluated stiffness of the VL in healthy subjects; therefore, future research should be carried out on muscular pathologies. Nevertheless, the information we provide will be helpful to future studies on myopathies to ensure reliable acquisition.

## 5. Conclusions

In conclusion, SWE and SE are reliable tools for evaluating the stiffness of VL, and a positive correlation was found between both types of elastography despite differences in their measurement principles. Although MyotonPRO and TMG measurements exhibited excellent intra- and inter-day reliability values, no correlation was described between MyotonPRO, TMG and elastography exams—SWE and SE—which could suggest that the MyotonPRO and TMG assess a different stiffness from elastography techniques. In addition, MyotonPRO exams are affected by superficial tissue thickness, so future studies should establish the maximal thickness of suprajacent tissue that does not influence myotonometry exams. Authors should discuss the results and how they can be interpreted from the perspective of previous studies and of the working hypotheses. The findings and their implications should be discussed in the broadest context possible. Future research directions may also be highlighted.

## Figures and Tables

**Table 1:** Mean values of Vastus Lateralis mechanical properties (Mean ± SD).

	Set 1	Set 2	Set 3	P_2-1_	P_3-1_	ICC_2-1_ (95%CI)	ICC_3-1_ (95%CI)	MDC_2-1_	MDC_3-1_
Shear Wave Elastography									
Shear Modulus (kPa)	16.16 ± 9.56	15.74 ± 10.33	17.49 ± 12.29	0.623	0.375	0.80 (0.68, 0.88)	0.62 (0.41, 0.77)	8.80	15.52
Strain Elastography									
Elastography Index (A.U.)	2.39 ± 0.51	2.42 ± 0.47	2.42 ± 0.49	0.762	0.922	0.71 (0.57, 0.81)	0.71 (0.57, 0.81)	0.73	0.75
MyotonPRO									
Dynamic Stiffness (N/m)	321.45 ± 56.89	322.65 ± 58.15	318.19 ± 58.24	0.555	0.311	0.97 (0.95, 0.98)	0.93 (0.89, 0.96)	28.37	40.38
Tensiomyography									
Radial Displacement (mm)	6.71 ± 1.83	6.70 ± 1.88	6.66 ± 1.74	0.946	0.744	0.91 (0.85, 0.95)	0.89 (0.82, 0.94)	1.48	1.56

## Data Availability

The data presented in this study are available on request from the corresponding author. The data are not publicly available due to restrictions of the subjects’ agreement.
